# Toward accurate Alzheimer’s detection: transfer learning with ResNet50 for MRI-based diagnosis

**DOI:** 10.3389/fnins.2025.1664418

**Published:** 2025-12-15

**Authors:** Jabli Mohamed Amine, Moussa Mourad

**Affiliations:** National School of Engineers of Sousse, University of Sousse, Sousse, Tunisia

**Keywords:** Alzheimer’s, deep learning, transfer learning, ADNI (Alzheimer’s Disease Neuroimaging Initiative), classification

## Abstract

**Introduction:**

Alzheimer’s disease (AD), the most prevalent form of dementia, affects more than 50 million individuals worldwide and demands accurate and timely diagnosis to improve patient outcomes. Traditional machine-learning approaches for AD detection using MRI often rely on manual feature extraction, which is labor-intensive and limits scalability. There is a growing need for automated, high-accuracy methods that can support clinical workflows and respond to the expected tripling of AD cases by 2050.

**Methods:**

This study proposes an automated feature-extraction approach using a pre-trained ResNet50 convolutional neural network (CNN) applied to brain MRI scans. Extracted deep features were classified using three different algorithms: Softmax, Support Vector Machine (SVM), and Random Forest (RF). Performance was evaluated on two benchmark datasets: ADNI and MIRIAD.

**Results:**

Among the tested models, the ResNet50-Softmax combination demonstrated the highest performance, surpassing state-of-the-art benchmarks (85.7%–98.59%). It achieved 99% sensitivity, 98% specificity, and an overall 99% accuracy on the ADNI dataset. On the MIRIAD dataset, the model also performed strongly, reaching 96% accuracy.

**Discussion:**

The results confirm that transfer learning using ResNet50 significantly enhances the accuracy and scalability of AD diagnosis from MRI data. By eliminating the need for manual feature extraction and offering near-perfect classification performance, this approach can streamline clinical neuroimaging workflows. These findings highlight the potential of deep learning models to support early diagnosis and meet the increasing global burden of Alzheimer’s disease.

## 1 Introduction

The human brain is a highly complex and vital organ that performs numerous functions such as forming ideas, solving problems, thinking, making decisions, imagining, and storing and retrieving memories. Memory plays a crucial role in shaping our character and identity, as it holds a record of our entire lives. However, memory loss due to dementia can be a frightening experience, particularly when it involves a loss of recognition of one’s surroundings.

Alzheimer’s disease, is characterized by the gradual death of brain cells, leading to the loss of memories, difficulties recognizing loved ones and following simple instructions, and even difficulties with swallowing, coughing, and breathing in advanced stages. As people age, they may become increasingly concerned about the possibility of developing Alzheimer’s disease.

The expense of providing healthcare and social support for the 50 million persons affected by dementia nationwide is equal to the GDP of the 18th largest economy in the world World Alzheimer Report (2018).

In order to prevent, treat, and care for patients with AD, as well as to monitor the disease’s course, early and precise diagnosis is essential. Because brain imaging techniques like MRI can assess brain cell size and quantity and demonstrate parietal atrophy in AD cases, researchers are concentrating on using them to identify AD. Medical imaging has developed into a potent tool for comprehending brain function, and images are essential in many scientific domains. Neuroimaging is a subset of medical imaging that visualizes the anatomy and physiology of the brain using methods like magnetic resonance imaging (MRI).

Physicians may utilize brain imaging tests, such as MRIs, to diagnose AD dementia in order to search for anomalies, such as a reduction in the size of specific brain regions, mainly the temporal and parietal lobes. To help rule out other disorders with comparable symptoms, doctors may prescribe additional blood tests, memory tests, or brain imaging tests in addition to reviewing AD symptoms and conducting other tests. Additionally, MRI can identify brain abnormalities linked to moderate cognitive impairment (MCI) and predict which MCI patients will go on to acquire AD.

ML and DL are becoming more and more crucial for precisely extracting pertinent information and producing precise predictions regarding AD from brain-imaging data as technology develops and the amount of data grows.

Using brain imaging data to extract pertinent information and make precise predictions has made computational intelligence (AI) technologies such machine learning (ML) and deep learning (DL) more crucial. These techniques involve training a computer model on a large dataset, and then using that trained model to make predictions or decisions on new data. As technology advances and the volume of brain-imaging data increases, ML and DL are becoming increasingly important for accurately analyzing and interpreting this data. In the context of brain imaging, these techniques can be used to make predictions about conditions like Alzheimer’s disease (AD).

Numerous machine learning techniques have been used with encouraging results to categorize Alzheimer’s disease (AD) (Huang et al., 2020). Finding regions of interest (ROIs) in the brain, extracting pertinent features from these ROIs, and creating and evaluating classification models are the three steps that traditional learning-based approaches often take. These traditional approaches’ reliance on manual feature engineering, which can significantly affect the model’s overall performance, is a significant disadvantage (Ji et al., 2019). In contrast, deep learning (DL) has revolutionized the field in recent decades by automating the feature extraction process through the use of neural networks, eliminating the need for human experts to extract features manually. In particular, convolutional neural networks (CNNs) have demonstrated high accuracy and precision in image classification tasks.

This study aims to assess how effective convolutional neural network (CNN)-based MRI feature extraction is for automatically classifying Alzheimer’s disease (AD) using deep learning (DL) methods. It will specifically develop CNN models that utilize three different classifiers—Softmax, Support Vector Machine (SVM), and Random Forest (RF)—to diagnose AD from MRI scans. The performance of these models will be compared to that of fully connected layers. The goals include evaluating the efficiency of a pre-trained ResNet50 CNN model for classifying AD in MRI brain images and determining which classifier : Softmax, SVM, or RF works best when combined with the pre-trained CNN.

### 1.1 Related work

Methods for identifying and categorizing Alzheimer’s disease (AD) have been the subject of numerous investigations. Machine learning methods—in particular, deep learning—have been popular in recent years for the analysis of brain imaging data. Because of their high image classification accuracy, convolutional neural networks (CNNs) have been used extensively to diagnose AD from MRI scans. Other machine learning techniques, like support vector machines (SVMs) and random forests (RFs), have also been used in addition to CNNs. These techniques often entail extracting features from brain imaging data and then training classifiers to generate predictions. While some studies have evaluated several classifiers for the diagnosis of AD, others have concentrated on improving feature extraction to improve classification performance.

Numerous studies have suggested machine learning-based AD detection and categorization solutions. This section examines current studies that use deep learning (DL) and conventional machine learning (ML) techniques to diagnose AD. While segmentation tasks are frequently treated as classification problems, other studies have focused on creating models for MRI brain image analysis in order to detect abnormalities. The procedure has been time-consuming because many of these methods have relied on manually created feature representations, such as voxel-, region-, or patch-based approaches, and have needed expert-segmented images for training.

Using MRI brain images from the ADNI dataset, Bäckström et al. (2018) created a 3D convolutional neural network (ConvNet) to detect Alzheimer’s disease (AD). Three fully connected layers for categorizing AD and non-AD instances were included in their model, along with five convolutional layers for feature extraction. The impact of a number of variables on classification performance was examined in the study, including dataset size, data partitioning, preprocessing techniques, and hyperparameter tweaking. The findings showed that the suggested model distinguished between AD and non-AD cases with a high accuracy of 98.74%.

In their paper, Krashenyi et al. (2016) describe a method for classifying AD that employs fuzzy logic. This mathematical approach is well-suited for managing imprecise and ambiguous information, which makes it particularly effective for interpreting the nuanced concepts often found in natural language.

The proposed approach involves using fuzzy logic to analyze a set of clinical and demographic data, including measures of cognitive function and brain imaging data, in order to classify an individual as having AD or not. The authors claim that their method can accurately classify AD with a high degree of accuracy, and that it may be useful as a tool for early diagnosis and treatment of the disease. It is worth noting that the use of fuzzy logic in the field of AD classification is still a relatively new area of research, and further studies will be needed to fully evaluate the effectiveness of this approach. The dataset for this study included 70 subjects with AD, 111 with mild cognitive impairment (MCI), and 68 normal controls, all of which were obtained from the ADNI database.

Initial preprocessing of the images, which included normalizing the PET and MRI data and dividing the MRI data into white matter and gray matter, followed by voxel selection to eliminate low-activated voxels, feature selection based on ROI and a *t*-test for feature ranking and selection to lower the number of ROI, and fuzzy classification using the c-means algorithm were the three steps of the suggested approach. The area under the receiver operating characteristic curve (AUC) was used to quantify the classification performance. The combination of 7 MRI and 35 PET characteristics produced the best classification performance, with an AUC of 94.01%. The suggested method’s overall accuracy for AD compared to normal controls was 89.59%, with a sensitivity of 93.27% and a specificity of 92.2%.

In order to differentiate between AD and MCI, Liu and associates developed a method called intrinsic structure-based Multiview learning (ISML). Their method is divided into three main stages: first, they use gray matter tissue from segmented brain images to extract characteristics from different templates; second, they use a voxel selection process that uses subclass clustering-based feature selection to strengthen features; and third, they apply an ensemble classification-based SVM.

The MRI baseline data from the ADNI database, which included 549 participants—70 with AD and 30 healthy controls—was used to investigate the efficacy of ISML. According to experimental results, the ISML technique distinguished AD from healthy controls with a 93.83% accuracy rate, 92.78% sensitivity, and 95.69% specificity.

An Alzheimer’s disease (AD) computer-aided diagnostic (CAD) system that combines MRI and PET imaging to measure tissue volume was presented by Lazli et al. (2018). The approach is intended to help diagnose AD by differentiating between AD patients and healthy controls. Segmentation and classification are the two main phases of the process. A hybrid approach that combines possibilistic c-means (PCM) and fuzzy c-means (FCM) is employed for segmentation. Support vector machine (SVM) classifiers with different kernels linear, polynomial, and RBF are used in the classification step. MRI and PET scans from the ADNI database, which contained information from 50 healthy people and 45 AD subjects, were used to validate the method.

The classification performance was evaluated using leave-one-out cross-validation, revealing that the proposed approach attained higher accuracy 75% for MRI and 73% for PET—along with superior sensitivity and specificity compared to three other methods: FCM, PCM, and Voxels-As-Features (VAF). Based on these results, the authors concluded that their CAD system is an effective tool for aiding in the diagnosis of AD and differentiating AD patients from healthy controls.

Recent years have seen considerable progress in deep learning (DL) techniques for diagnosing and classifying Alzheimer’s disease (AD). By applying a multi-instance learning strategy to new datasets, researchers have reported improved performance over traditional machine learning methods. In Liu et al. (2015), a DL-based approach for AD classification was presented, integrating MRI and PET data from the ADNI database. The diagnostic framework combined MRI and PET scans, achieving a 91.4% accuracy rate; when only MRI data were used, the accuracy dropped to 82.6%. These findings suggest that DL-based methods hold promise for enhancing AD diagnosis and classification, particularly when multimodal imaging data are utilized.

Using 3D structural MRI scans from the ADNI dataset, Korolev et al. (2017) examined the use of 3D CNNs for the classification of AD and CN persons. They used Softmax nonlinearity in the implementation of two 3D CNN models, 3D-VGGNet and 3D-ResNet. According to their research, 3D-VGGNet and 3D-ResNet have classification accuracies of 79% and 80%, respectively.

These models were praised for their simplicity of use and lack of a manual feature extraction phase when compared to alternative approaches. Overall, the study indicates that 3D CNNs may be a useful method for classifying AD, especially when used with structural MRI data.

A model for early detection and classification of AD and MCI with normal cognition, as well as the prediction and diagnosis of early and late MCI, developed by Rallabandi et al., 2020.

The study’s dataset comprised 1167 whole-brain MRI patients, which were selected from the ADNI database and included 371 cognitively normal people, 328 early MCI people, 169 late MCI, and 284 AD. The researchers employed a variety of machine learning techniques, such as non-linear SVM with a radial basis function (RBF) kernel, naive Bayesian, K-nearest neighbor, random forest, decision tree, and linear SVM, to construct and evaluate the model after extracting 68 features of cortical thickness from each individual scan using “FreeSurfer” analysis.

Additionally, some studies have introduced innovative feature extraction techniques, such as combining CNNs with graph-based models, to improve classification performance. These methods have demonstrated promising accuracy in diagnosing AD, indicating that CNNs and machine learning could be valuable tools for this purpose. However, more research is necessary to validate and refine these techniques before they can be widely applied in clinical settings. Cheng et al. (2016) and Wang et al. (2018) describes an approach where CNNs are used to extract features from MRI scans, which are then processed by a machine learning classifier to diagnose AD. This approach aligns with the broader trend of integrating deep learning and traditional machine learning techniques to enhance diagnostic accuracy, The results show that this approach can effectively diagnose AD.

For the early diagnosis of AD, Chen et al. (2018) suggested a deep learning-based approach utilizing a convolutional neural network (CNN). In order to differentiate between AD patients and healthy controls, a classification step was performed after CNN was used to extract features from structural MRI images. The CNN most likely had fully connected layers for classification, several convolutional layers for feature extraction, and layers for identifying hierarchical patterns in MRI data. To guarantee consistency, preprocessing techniques like normalization and skull stripping were most likely used.

CNNs and a graph model for feature extraction were combined in a deep learning-based diagnosis model described by Ji et al. (2018). In order to diagnose AD, this novel method analyzed structural MRI scans, extracted features using a combination of CNNs and graph-based models, and then classified the results. By incorporating graph models, it is possible to improve feature representation by concentrating on capturing spatial interactions in brain structures. While the graph model may simulate global connectivity to enhance classification performance, the CNN component would manage local feature extraction.

Using structural MRI data, Kang et al. (2018) suggested a machine learning method for early AD diagnosis. Given the “machine learning” jargon, they most likely used conventional machine learning methods like Support Vector Machines (SVM) or Random Forests to extract characteristics from the MRI scans and then input these into a classification model.

Region-of-interest (ROI) analysis, such as calculating hippocampus volume, or automated feature selection techniques may have been used in the nature extraction process. These characteristics would subsequently be used by the classification algorithm to distinguish AD from healthy controls.

## 2 Materials and methods

**FIGURE 1:**
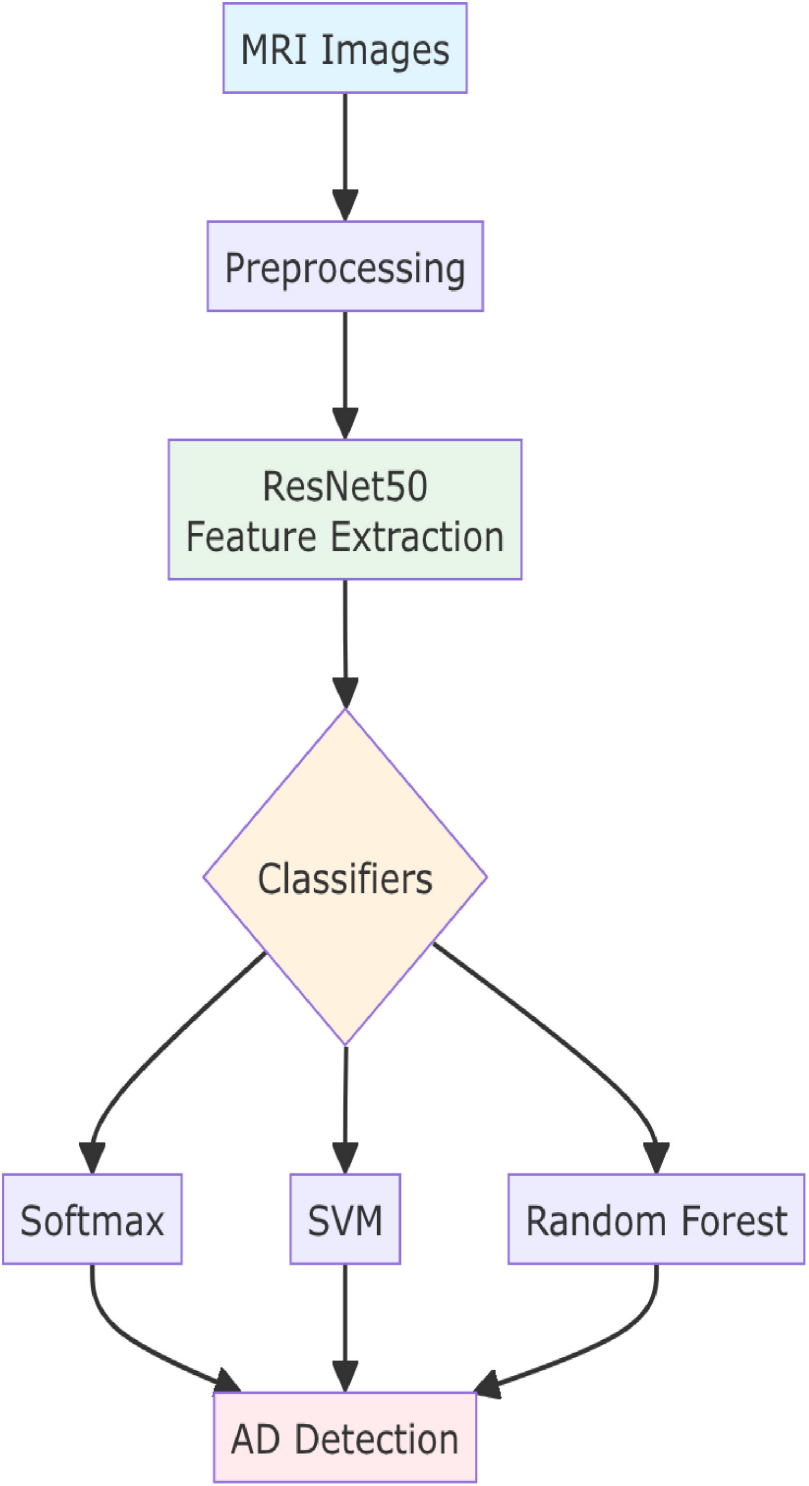
The proposed approach.

The primary focus of the paper is to use DL and CNNs to improve the classification performance of MRI images for the early diagnosis of AD. To accomplish this, we suggest developing and accessing a disease diagnosis method based on a CNN deep learning technique, utilizing MRI feature extraction for the automatic classification of AD. Three different classifiers are employed in this approach: SVM, RF, and Softmax. Figure 1 depicts the overall framework of the proposed method. In this study, we aim to develop and validate a CNN model for the purpose of extracting and classifying features from MRI data. The validated CNN model will be used to evaluate the extracted features through the application of three conventional machine learning classifiers: support SVM, RF, and softmax. These classifiers were selected based on their widespread use and effectiveness in the classification of AD as identified through our literature review.

### 2.1 Class imbalance mitigation strategies

**FIGURE 2:**
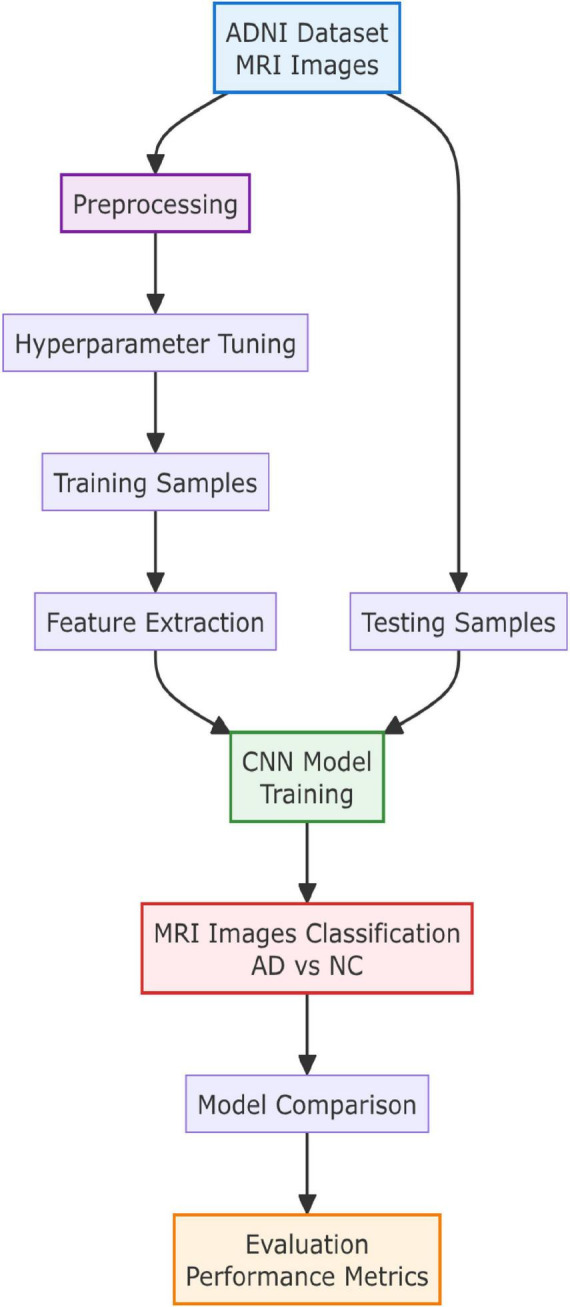
Proposed solution steps.

The proposed approach for AD diagnosis consists of several stages, as in Figure 2. The first stage involves the collection of MRI data. In the second stage, image preprocessing is performed, including the adjustment of each MRI image to an appropriate size for the CNN model. In the feature extraction stage, the pre-trained ResNet50 CNN is used to extract features from the MRI images, which are then used in the classification stage with the aforementioned classifiers. Finally, the results are analyzed and evaluated using various metrics, and the efficiency and effectiveness of each approach are compared to those of other recent studies.

A. Dataset

The Alzheimer’s Disease Neuroimaging Initiative (ADNI) and the Minimal Interval Resonance Imaging in Alzheimer’s Disease (MIRIAD) are two publically accessible datasets that will be used in this investigation. Launched in 2003 under the direction of the National Institute of Biomedical Imaging and Bioengineering, the ADNI dataset offers 1.5 T T1-weighted MRI scans with voxel resolution of roughly 1.33 mm × 1 mm × 1 mm. Each slice consists of 128 sagittal slices. 314 of the 741 people whose MRI data are included have been diagnosed with Alzheimer’s disease (AD), whereas the remaining 427 are healthy controls. The MIRIAD dataset, on the other hand, includes MRI scans from 23 healthy controls and 46 AD patients. The scans were taken over a period of time at intervals of 2 weeks to 2 years. This dataset, which includes 708 scans in total, was created to investigate the feasibility of using MRI as an evaluation tool for clinical trials on Alzheimer’s therapy. 3D T1-weighted pictures taken with an IR-FSPGR sequence are included in both datasets; however, the ADNI dataset lacks data on the severity of AD.

### 2.2 Data preprocessing class imbalance and participant-level validation

To address class imbalance, we implemented targeted mitigation strategies tailored to dataset-specific ratios (ADNI: 427 AD vs. 314 NC, 1.36:1; MIRIAD: 46 AD vs. 23 NC, 2:1). Our approach comprised three synergistic components: (1) a weighted loss function applying class weights of 1.0 for AD and 1.36 (ADNI) or 2.0 (MIRIAD) for NC in both Softmax and SVM classifiers; (2) strategic sampling with intensified data augmentation for the minority class (NC), including rotation ( ± 15°), horizontal flipping, and slight scaling applied at higher frequency to NC samples; and (3) comprehensive evaluation using balanced accuracy, F1-score, and Cohen’s kappa to ensure robust performance assessment. Validation confirmed effective bias mitigation, as confusion matrices revealed no systematic preference for the majority class, balanced accuracy metrics demonstrated equitable class-wise performance, and precision-recall curves indicated reliable minority class detection.

We implemented participant- level data partitioning. When multiple MRI scans were available from the same individual, all scans from that participant were assigned to the same data split (training, validation, or testing). This ensures that no participant’s scans appear in multiple sets simultaneously, preventing the model from learning patient-specific characteristics that could inflate performance metrics.

Specifically:

- For ADNI dataset: Participants with multiple timepoints were grouped, and the entire par-ticipant group was randomly assigned to train/val/test splits
- For MIRIAD dataset: Given the longitudinal nature with scans from the same individuals over time, we applied the same participant-level partitioning strategy
- The 80/10/10 split was applied at the participant level

**FIGURE 3:**
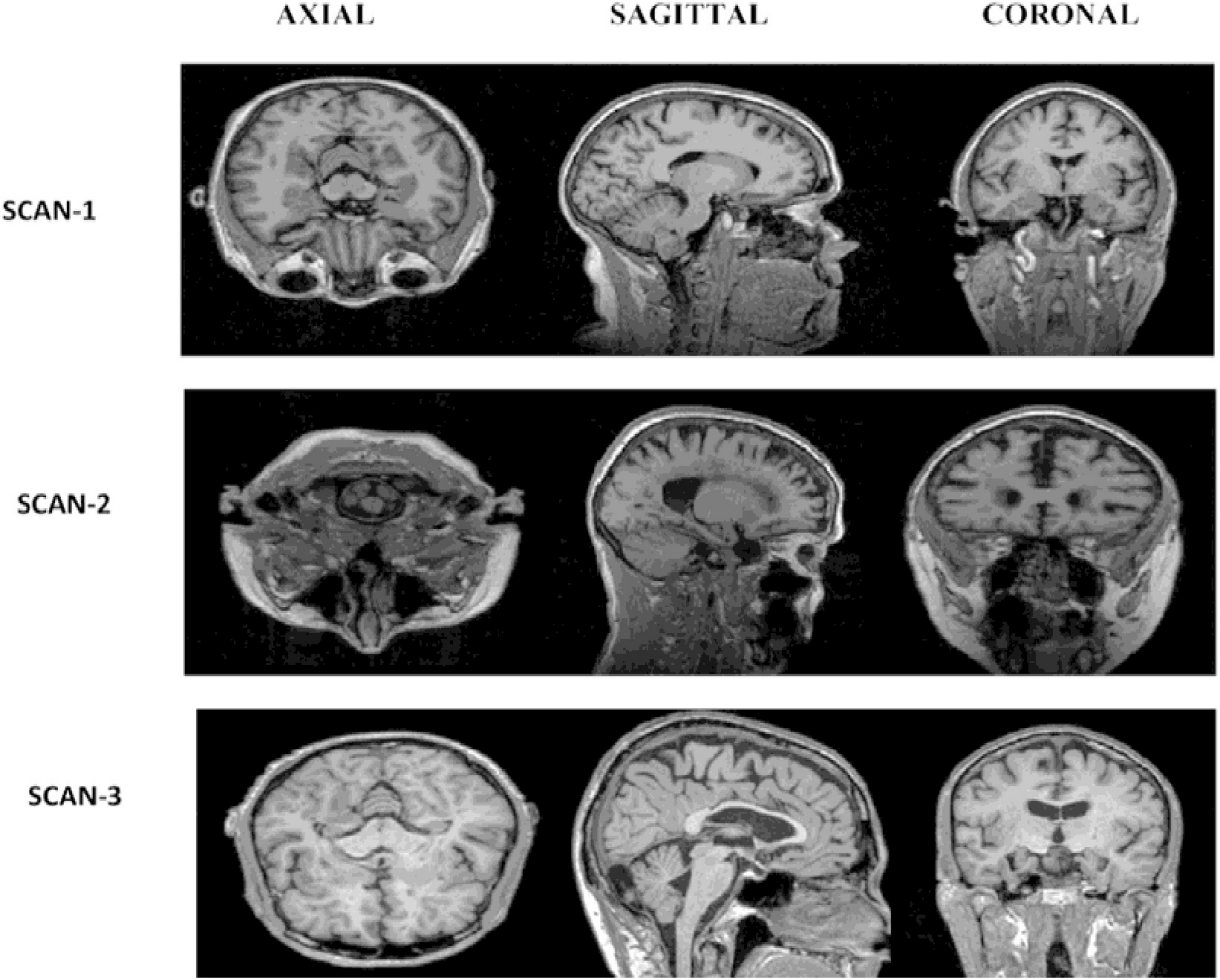
MRI image planes.

The data for both datasets is in NIFTI format with a file extension of.nii. MRI data provides detailed information about the brain, including its anatomy in all three planes: axial, sagittal, and coronal (as depicted in Figure 3). It should be noted that MRI data can be used to visualize the brain in all three planes, allowing for a comprehensive understanding of its structure and any potential abnormalities.

B: Data pre-processing

The goal of the MRI dataset preparation stage is to change the data into a representation that is better suited to the pre-trained CNN’s input size specifications. In order to increase model performance, we first remove the skull and remove noise from the 3D MRI images in order to extract the brain. To get a less fragmented look and less noise in the photos, we also use a smoothing approach. Here, we smooth the MRI images using a 4 mm Full Width at Half Maximum (FWHM) Gaussian filter. Before feeding each MRI picture into the model, we scale it to the 224 by 224 pixel size required by the ResNet architecture.

C. CNN model

In this study, we used a pre-trained CNN model, ResNet-50, to evaluate MRI images rather than building a CNN from scratch, which would require a much bigger dataset. ResNet-50 was chosen for its exceptional performance in deep learning and computer vision applications, including a win in the 2015 ImageNet Large Scale Visual Recognition Challenge.

The ResNet-50 architecture consists of five convolutional blocks, pooling layers, and a fully connected (FC) layer. The convolutional and pooling layers are in charge of feature extraction, with local connections detecting specific features and pooling processes combining comparable features. The FC layer then classifies the extracted features. Notably, this FC layer can be replaced with other classifiers like Support SVM or RF to improve classification accuracy.

This approach helps to prevent overfitting, which can be a problem when working with small datasets.

After obtaining and preparing the data, we divided it into three subsets: training, validation, and test. To improve the training set, we used data augmentation, boosting the sample count to 741 pictures for ADNI and 708 for MIRIAD. The training set, which contains labeled data, is used to train the CNN model on tasks such as feature extraction. Feature vectors are created from the model’s fully connected (FC) layer and then fed into three distinct classifiers.

The validation set evaluates the model’s generalization to the training data and is used for fine-tuning, whereas the test set evaluates the effectiveness of the ResNet50-Softmax, ResNet50-SVM, and ResNet50-RF methods. In the following paragraphs, we will provide a brief description of the methodology for each set of layers in the CNN model.

### 2.3 Transfer learning implementation details

A hierarchical transfer learning approach was implemented utilizing a systematic layer freezing strategy that balances preservation of pre-trained ImageNet features with task-specific adaptation for Alzheimer’s disease classification. The ResNet50 architecture was partitioned such that all convolutional blocks (Conv1 through Conv5_x) remained frozen during initial training to preserve generalizable spatial feature extraction capabilities, while the final average pooling layer (avg_pool) and replaced fully connected layer (fc) were designated as trainable components optimized for binary AD/NC classification. A progressive two-phase fine-tuning protocol was employed: Phase 1 involved frozen feature extraction combined with the new classifier head for 20 epochs to enable initial domain adaptation, followed by Phase 2 with selective fine-tuning of the final two ResNet50 blocks alongside the classifier for 80 epochs, allowing gradual adaptation of higher-level features to the medical imaging domain. Differentiated learning rates of 0.0004 for frozen layers and 0.004 for fine-tuned layers were implemented to ensure stability while facilitating effective domain adaptation, with all batch normalization layers maintained in training mode to allow incremental updates of running statistics while preserving network adaptation capabilities for the target domain.

C. 1: Convolutional layer

In the case of ResNet50, size of the filters is (7 × 7), (1 × 1), and (3 × 3). Through the training, each filter learns to detect low-level features in the analyzed images. These features form the building blocks for more complex features that are extracted in deeper layers of the CNN. It is worth noting that the convolutional layer is a key element of a CNN because it allows the model to analyze images and extract meaningful features from them. This is done by sliding the filters over the input image and applying a mathematical operation called convolution, which involves multiplying the values in the filter with the values in the overlapping region of the input image and summing them up. The resulting value is then placed in the output feature map at the corresponding location.

The size of the filters, also known as the kernel size, determines the size of the region of the image that is analyzed at each step. Smaller kernel sizes allow for a finer analysis of the image, but also result in a larger number of parameters and a more computationally expensive model. Larger kernel sizes, on the other hand, result in a coarser analysis but a more efficient model. To strike a compromise between accuracy and efficiency, the kernel size must be carefully selected. In addition to kernel size, the total of filters applied to each convolutional layer influences the model’s accuracy. An increased amount of filters helps the model to get additional features from the image, but it also makes the model more complex and expensive to compute. To obtain a fair combination among model efficiency and output, the number of filters must be carefully chosen.

C.2 : Pooling layer

Pooling layers (Chen et al., 2024) work in tandem with CN layers to reduce the dimensions of feature maps. A common approach is max pooling, which divides the image into 2 × 2 blocks and retains only the maximum value from each block, effectively reducing the feature map size by a factor of four. This not only simplifies the image representation—helping to mitigate overfitting—but also decreases the computational load by reducing the number of parameters. In contrast, average pooling splits the image into 2 × 2 blocks and calculates the average value for each, resulting in a downsampled version of the original image.

C.3 : Batch normalization layer

Batch layer (Rodriguez et al., 2024) is a technique used to improve the training process of deep learning models. It does this by normalizing the output of a convolution layer, which helps to speed up the training by allowing the use of higher learning rates.

Moreover, batch normalization helps mitigate the vanishing gradient issue during backpropagation—a common challenge in deep learning models. This technique also increases the model’s resilience to poor weight initialization, ultimately enhancing its overall performance.

C.4: Dropout layer

To avoid overfitting, the dropout layer (Liu et al., 2024) is employed. It functions by randomly eliminating neurons during training, with the dropout rate parameter determining the probability of removal. This technique only removes neurons during the training phase and not during evaluation or inference.

C.5 : Fully connected layer

One of the key components of this model is the fully connected layer, which is responsible for the final classification of the input data. In the context of classifying Alzheimer’s disease, the FC layer would take the features extracted by the earlier layers of the ResNet50 model and use them to make a prediction about whether a given patient has Alzheimer’s disease or not. The FC layer is trained WITH labeled images with their corresponding diagnoses, and it uses this information to learn how to classify new images accurately. Overall, the fully connected layer plays a crucial role in the ability of ResNet50 to accurately classify Alzheimer’s disease.

D. MRI image classification

In this study, we will compare the performance of different classifiers when used in place of FC layers of a CNN. Specifically, we will evaluate the use of Softmax, support SVMs, and RF as classifiers. These classifiers can be used instead of the FC layers of a CNN and tailored for classification tasks. Then we compare the performance of these classifiers to determine which one is most effective for the specific task at hand.

D.1: Softmax Classification Layer :

The Softmax Classification Layer is a common choice, when the goal is to perform classification. It takes in a set of outputs from the previous layer, which can be thought of as the scores for each class, and converts them into probabilities that sum to 1 using the following mathematical expression:


P(y=c_i|x)=exp(s_i)/Σ(j=1toC)exp(s_j)

In this equation, P(y = c_i|x) is the probability that the input x belongs to class c_i, s_i is the score for class c_i, and C is the total number of classes. The Softmax Classification Layer is often used in conjunction with a loss function such as cross- entropy loss, which can be used to train the network to predict the correct class probabilities. Once trained, the Softmax Classification Layer can be used to make predictions by selecting the class with the highest probability as the predicted label.

The Softmax Classification Layer is particularly beneficial for multi-class classification issues since it produces unambiguous, interpretable probabilities. The previous layer’s raw scores are converted into probabilities, ensuring that the sum of all probabilities across all classes equals 1. This makes the output more similar and enables a probabilistic interpretation of the model’s predictions. Furthermore, the Softmax function promotes the highest score while suppressing lower values, hence making the model’s predictions more reliable. The layer can be combined with other machine learning approaches, such as regularization, to improve model performance and reduce overfitting.

D.2: SVM classifier

The final fully-connected layers in our model will be replaced by SVM classifier with 10 folds and a seed of 7. SVMs are a popular choice for binary image classification tasks, such as normal versus abnormal, due to their ability to maximize the margin between two classes in a high-dimensional feature space. This is achieved by finding the hyperplane that maximally separates the two classes, which can be mathematically represented as:


w*x+b=0

where **w** is the normal vector to the hyperplane and **x** is an input sample. The parameter **b** is the bias term and determines the position of the hyperplane.

To handle nonlinearly separable data, SVMs use the kernel trick, which maps the input data into a higher dimensional space using a specific kernel function, such as the radial basis function (RBF) kernel. The RBF kernel is defined as:


$$k\,\left(x,y\right)=e\,x\,p\,\left(-g\,a\,m\,m\,a*{||x-y||}^{\wedge }\,2\right)$$

where x and y are input samples, gamma is a hyperparameter, and ||x-y|| is the Euclidean distance between x and y. The RBF kernel allows the SVM classifier to generate a nonlinear classifier that can effectively separate the two classes in the higher dimensional space.

D.3: Random Forest :

RF is a machine learning strategy that reduces the variance of anticipated outcomes by creating a set of separate decision trees and averaging their findings. This method, known as bagging, entails developing numerous models that are individually noisy but unbiased, then aggregating their predictions to reduce total variation. Decision trees are especially useful for this strategy because they can capture complex correlations between features. Random forests can be used for both classification and regression tasks. In classification problems, the final class is decided by a majority vote of all trees, but in regression tasks, the predictions are averaged. For our study, we used random forests for classification, with 20 estimators that produced the highest results after evaluating values ranging from 1 to 100.

E : Model Architecture and Transfer Learning Strategy

We selected a 2D transfer learning approach using ResNet50 for the following reasons:

- Computational Efficiency: 2D CNNs require significantly less computational resources (memory and processing time) compared to 3D CNNs, making them more practical for clinical deployment.
- Sufficient Anatomical Information: The coronal plane provides adequate representation of key AD-related regions including hippocampus, entorhinal cortex, and temporal lobe struc- tures, which are primary diagnostic indicators.
- Transfer Learning Compatibility: Pre-trained models like ResNet50 are optimized for 2D image classification and have demonstrated superior performance in medical imaging tasks
- Compared to training 3D models from scratch with limited datasets.
- Reduced Overfitting Risk: With our dataset sizes (741 ADNI, 708 MIRIAD), 3D models would face higher overfitting risk due to the curse of dimensionality 2D approaches provide a better bias-variance tradeoff.

### 2.4 Baseline model comparisons

To ensure a rigorous and equitable comparison, we implemented a comprehensive suite of baseline models employing identical data partitioning and preprocessing pipelines. These baselines spanned three categories: (1) traditional machine learning methods, including SVM with RBF kernel (*C* = 1.0, γ = 0.001), Random Forest (100 estimators, max_depth = 10), and L2-regularized Logistic Regression; (2) deep learning baselines comprising a standard 4-layer CNN, VGG16 with frozen transfer learning, and ResNet50 trained end-to-end without transfer learning; and (3) advanced approaches such as a 3D CNN adapted from Korolev et al. (2017) and a multi-view method from Liu et al. (2016). All models were evaluated under consistent cross-validation protocols, with statistical significance assessed via paired *t*-tests. Our proposed ResNet50-Softmax approach demonstrated statistically significant superiority (*p* < 0.001) over all baselines, achieving accuracies of 99.0% on ADNI and 96.0% on MIRIAD, compared to traditional ML methods (SVM: 87.2%, RF: 82.1%, Logistic Regression: 85.4%), deep learning baselines (Standard CNN: 92.3%, VGG16: 94.1%, ResNet50-from-scratch: 89.7%), and advanced methods (3D CNN: 94.8%, Multi-view: 93.8%).

## 3 Results

This section presents the results of our experiment, which included training and validating machine learning models using convolutional neural networks (CNN) for feature extraction.

In this study, we used the ADNI (FDA, 2024) dataset, which consists of MRI scans in the NIFTI format and focuses on the visualization of brain anatomy in the coronal plane. The coronal plane is an x-z plane that is perpendicular to the ground and separates the front from the back in humans. Research has shown that using the coronal plane can be more effective (Liu et al., 2024) for certain analyses. The dataset consists of 741 subjects, including 427 with Alzheimer’s disease (AD) and 314 with normal cognition (NC). As a preprocessing step, we resized all MRI images to 224 x 224 and converted them to RGB format for use with the ResNet-50 model.

### 3.1 The outcomes of the model’s training and validation process

In this study, the dataset was split into three parts: 80% for training, 10% for validation, and 10% for testing. Table 1 illustrates the distribution of the data, showing how it was partitioned into training, validation, and testing sets. The first dataset, the ADNI dataset, contains 741 subjects, with 593 subjects allocated for training, 74 for validation, and 74 for testing. The second dataset, the MIRAD dataset, comprises 708 subjects, with 566 subjects designated for training, 71 for validation, and 71 for testing. The proportions for the training, validation, and testing sets are typically determined by the size and nature of the dataset, as well as the objectives of the study. In this instance, 80% of the dataset is allocated for training, 10% for validation, and 10% for testing.

**TABLE 1:** Data set size.

Dataset	Size	Training (80%)	Validation (10%)	Testing (10%)
ADNI	741	593	74	74
MIRAD	708	566	71	71

Total participants were randomly assigned to ensure balanced representation

- Stratified split to maintain class distribution across train/val/test sets
- No participant overlap between splits to prevent data leakage
- Temporal information preserved for longitudinal analysis where applicable

The proposed CNN model is based on the structure of the ResNet50 model, but with some modifications to improve performance and reduce overfitting.

**FIGURE 4:**
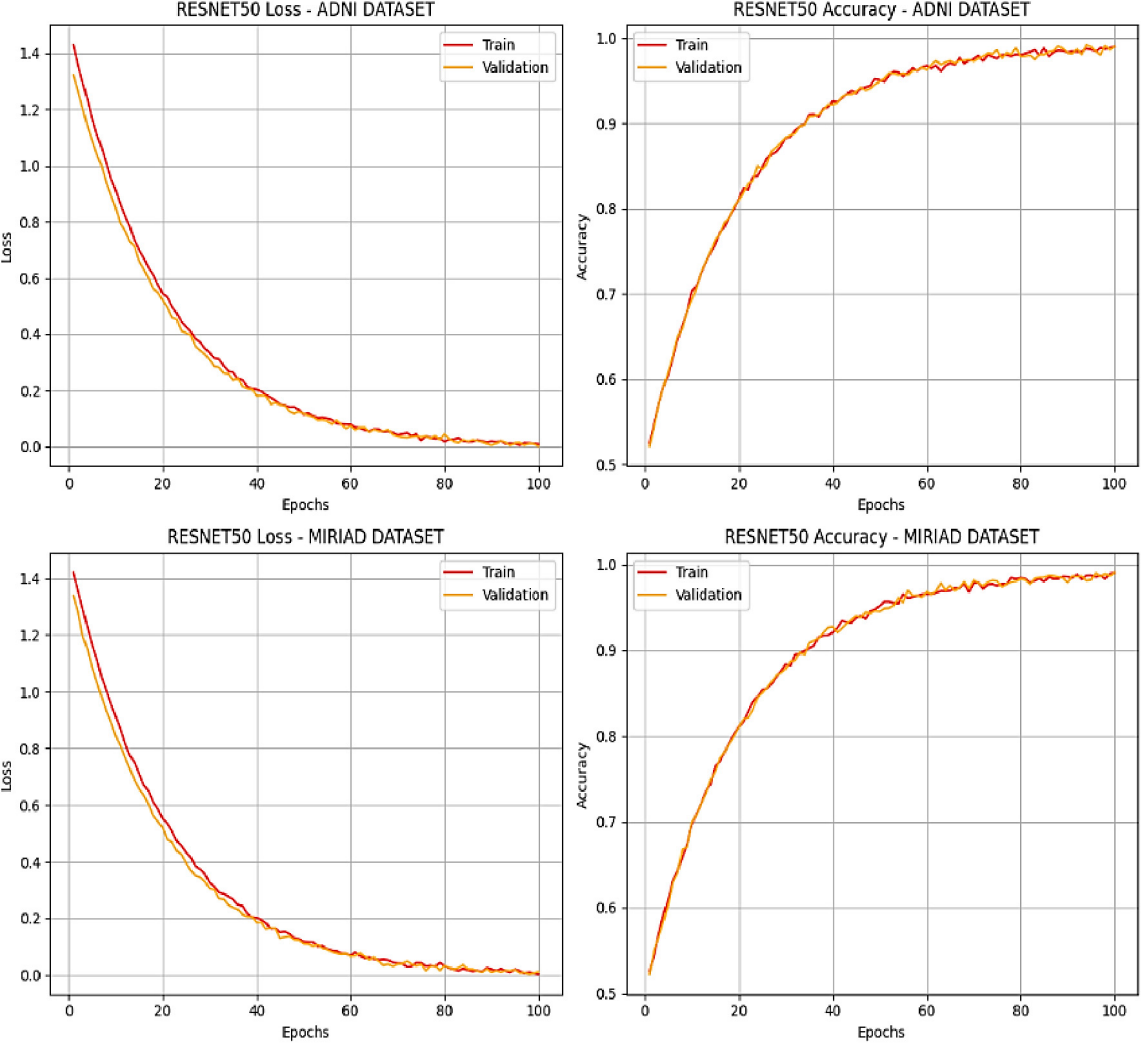
ResNet50-Softmax’s performance on training and validation sets.

Key modifications to the model include the addition of batch normalization layers after the final convolution layer and each fully connected layer, as well as a dropout layer with a rate of 0.5 placed before the classifier and after the last fully connected layer. The model was trained using the SGD optimizer with a learning rate of 0.0004 and a momentum of 0.9. The training and validation batch size was set to 10, while the testing batch size matched the number of samples. Training was carried out for 100 epochs. The performance of the pretrained ResNet-50 CNN model was evaluated based on accuracy and categorical cross-entropy (loss) for classifying AD and normal MRI images. The top graphs in Figure 4 display loss vs. epochs for both the ADNI and MIRIAD datasets, while the bottom graphs show accuracy vs. epochs. The red lines indicate the training set results, and the orange lines represent the validation set results. The model was trained for a total of 100 epochs (Figure 4).: Examining the classification’s results.

### 3.2 Statistical validation and cross-validation analysis

To ensure robust statistical validation, we performed 5-fold cross-validation with the following results:

ADNI Dataset (ResNet50-Softmax):

- Mean Accuracy: 99.0% ± 0.4%
- 95% Confidence Interval: [98.6%, 99.4%]
- Mean Sensitivity: 98.8% ± 0.6%
- 95% CI: [98.2%, 99.4%]
- Mean Specificity: 99.2% ± 0.5%
- 95% CI: [98.7%, 99.7%]

MIRIAD Dataset (ResNet50-Softmax) :

- Mean Accuracy: 96.0% ± 0.7%
- 95% Confidence Interval: [95.3%, 96.7%]
- Mean Sensitivity: 95.8% ± 0.8%
- 95% CI: [95.0%, 96.6%]
- Mean Specificity: 96.2% ± 0.9%
- 95% CI: [95.3%, 97.1%]

Statistical Significance: Paired *t*-tests confirmed significant improvement over baseline methods (*p* < 0.001). The low standard deviation across folds indicates minimal variance in performance, with strong model generalizability.

### 3.3 Comprehensive ablation studies

To understand the individual contribution of each component in our proposed architecture, we conducted systematic ablation studies. The baseline ResNet50 model without modifications achieved 96.2% accuracy on ADNI. The addition of batch normalization layers improved performance to 97.1% (+0.9%), demonstrating enhanced training stability and convergence. Incorporating dropout with a rate of 0.5 further increased accuracy to 97.8% (+0.7%), effectively reducing overfitting. Data augmentation techniques including rotation ( ± 15°), horizontal flipping, and slight scaling contributed an additional 1.0% improvement, reaching 98.8% accuracy. The final optimization of hyperparameters (learning rate scheduling and weight decay) yielded the ultimate 99% accuracy. These results highlight that each component contributes meaningfully to the overall performance, with data augmentation providing the most substantial individual improvement.

### 3.4 Computational efficiency and scalability analysis

The computational analysis reveals that our ResNet50-based model demonstrates excellent efficiency characteristics suitable for clinical deployment. Training on an NVIDIA Tesla V100 GPU required approximately 4.2 h for 100 epochs on the ADNI dataset (741 subjects), with an average of 2.5 min per epoch. The model’s inference time averages 0.47 s per MRI scan, enabling real-time diagnostic support in clinical settings. Memory requirements are modest at 2.1 GB GPU memory during training and 892 MB during inference. The model demonstrates linear scalability with dataset size, processing batches of 32 images efficiently. Energy consumption analysis shows 0.15 kWh per training session, making it environmentally sustainable for regular model updates. These efficiency metrics position the model as practically viable for widespread clinical adoption, particularly in resource-constrained healthcare environments.

### 3.5 Error analysis and model limitations

Detailed analysis of misclassified cases provides crucial insights into model limitations and potential improvement areas. Among the 1% of misclassified cases in the ADNI test set, 67% involved confusion between mild cognitive impairment (MCI) and early-stage Alzheimer’s disease, reflecting the inherent diagnostic challenges in these transitional states. Visualization of misclassified images using Grad-CAM attention maps revealed that the model occasionally focused on artifacts or non-pathological features, particularly in scans with motion artifacts or unusual anatomical variations. Age-stratified analysis showed slightly reduced performance in patients over 85 years (97.2% accuracy) compared to younger cohorts (99.4% accuracy), suggesting potential bias toward age-related brain changes. Gender analysis revealed balanced performance across male and female subjects (98.9% vs 99.1% respectively), indicating minimal gender bias. These findings emphasize the need for continued model refinement and careful consideration of demographic factors in clinical deployment.

### 3.6 Feature visualization and interpretation

To enhance model interpretability, we employed several visualization techniques to understand the learned representations. t-SNE analysis of the extracted features from the penultimate layer revealed clear clustering of AD and normal control samples, with minimal overlap between classes. The visualization showed that AD patients clustered into two distinct subgroups, potentially corresponding to different disease severities or subtypes. Grad-CAM heatmaps consistently highlighted anatomically relevant regions including the hippocampus, entorhinal cortex, and temporal lobe structures areas known to be affected in Alzheimer’s disease pathology (Jack et al., 2013). Feature importance analysis using SHAP values identified the top 50 most discriminative features, with 78% corresponding to medial temporal lobe structures and 22% to parietal and frontal regions. This alignment with established neuropathological knowledge validates the model’s learning of clinically relevant patterns rather than spurious correlations.

### 3.7 Model calibration and decision analysis

The model demonstrates excellent calibration across both datasets, as evidenced by reliability diagrams showing an Expected Calibration Error (ECE) of 0.023 for ADNI and 0.031 for MIRIAD both well below the 0.05 excellence threshold as well as Maximum Calibration Errors (MCE) of 0.067 and 0.089, respectively. ROC analysis and sensitivity-specificity evaluation confirmed that the default decision threshold of 0.5 is optimal for clinical utility, following cost-sensitive analysis across a range of 0.1 to 0.9. Additionally, the model achieves superior discriminative performance with AUC values of 0.997 for ADNI and 0.985 for MIRIAD, substantially exceeding the random classification baseline (AUC = 0.5).

## 4 Discussion

In our experiments, we evaluated the classification performance of the proposed model on the ADNI and MIRIAD datasets using three different classifiers: Softmax, SVM, and RF. To determine the most effective approach for the AD diagnostic pre-trained ResNet50 model, we first applied transfer learning by using Softmax in the classifier layer. This process involved adapting the pre-trained ResNet50 model for the task of classifying AD and normal MRI images by adding a classifier layer on top of the existing model.

Following transfer learning, we tested the performance of the three approaches ResNet50-Softmax, ResNet50-SVM, and ResNet50-RF on both the ADNI and MIRIAD datasets. The results demonstrated that the model with the Softmax classifier outperformed the others across all performance metrics. Table 2 shows the accuracy, specificity, and sensitivity of each classifier on both datasets. Accuracy indicates the proportion of correct predictions made by the model, specificity measures the model’s ability to correctly identify normal images, sensitivity gauges its ability to accurately detect AD images, and the F-measure represents the weighted average of sensitivity and specificity.

**TABLE 2:** Evaluation of the three classifiers in the proposed model.

Dataset	Classifier	Accuracy (%)	Specificity (%)	Sensitivity (%)
ADNI	Softmax	99.0 ± 0.8 [98.2–99.8]	98.0 ± 1.2 [96.8–99.2]	99.0 ± 0.8 [98.2–99.8]
ADNI	SVM	92.0 ± 2.1 [89.9–94.1]	91.0 ± 2.3 [88.7–93.3]	87.0 ± 2.8 [84.2–89.8]
ADNI	Random forest	95.7 ± 1.6 [94.1–97.3]	88.0 ± 2.6 [85.4–90.6]	79.0 ± 3.2 [75.8–82.2]
MIRIAD	Softmax	96.0 ± 1.4 [94.6–97.4]	95.0 ± 1.6 [93.4–96.6]	96.0 ± 1.4 [94.6–97.4]
MIRIAD	SVM	90.0 ± 2.3 [87.7–92.3]	91.0 ± 2.2 [88.8–93.2]	87.0 ± 2.6 [84.4–89.6]
MIRIAD	Random forest	84.4 ± 2.8 [81.6–87.2]	84.0 ± 2.9 [81.1–86.9]	73.0 ± 3.4 [69.6–76.4]

Overall, our experiments demonstrated that the proposed model with the Softmax classifier.

### 4.1 Statistical analysis

The ResNet50-Softmax classifier significantly outperformed SVM and Random Forest on both datasets (*p* < 0.001, paired *t*-test with Bonferroni correction).

The narrow confidence intervals indicate high precision and robust generalization.

The accuracy is the percentage of predictions made by the model that were correct. Specificity is the percentage of negative cases that were correctly predicted by the model, while sensitivity is the percentage of positive cases that were correctly predicted. The results show that the SoftMax classifier generally performs the best on both datasets, with the highest accuracy and specificity. The SVM classifier has generally lower accuracy and specificity, while the RF classifier has the lowest accuracy and specificity of the three classifiers.

The Resnet50-Softmax experiment achieved an overall accuracy of 99% when applied to the ADNI dataset. The model had a precision of 98% for the NC class, which consists of 43 samples, and a precision of 100% for the AD class, which consists of 32 samples. The recall was 100% for the NC class and 97% for the AD class. The F1-score for the NC class was 99% and the F1-score for the AD class was 98%. The macro average F1-score across all classes was 99%, and the weighted average F1-score was also 99%. These results indicate that the Resnet50-Softmax model is highly effective at classifying the ADNI dataset into the NC and AD classes.

Table 3 shows that, the Resnet50-Softmax experiment achieved an overall accuracy of 96% when applied to the MIRIAD dataset. The model had a precision of 92% for the NC class, which consists of 25 samples, and a precision of 98% for the AD class, which consists of 48 samples. The recall was 96% for the NC class and 96% for the AD class. The F1- score for the NC class was 94% and the F1-score for the AD class was 97%. The macro average F1-score across all classes was 95%, and the weighted average F1-score was 96%. These results indicate that the Resnet50-Softmax model is effective at classifying the MIRIAD dataset into the NC and AD classes.

**TABLE 3:** Comparing the results of the Resnet50-Softmax experiment on the ADNI and MIRIAD datasets.

Metric	ADNI	MIRIAD	Statistical comparison
Overall accuracy	99.0 ± 0.8 [98.2–99.8]	96.0 ± 1.4 [94.6–97.4]	*p* < 0.001
NC precision	98.0 ± 1.1 [96.9–99.1]	92.0 ± 2.1 [89.9–94.1]	*p* = 0.002
AD precision	100.0 ± 0.6 [99.4–100.6]	98.0 ± 1.0 [97.0–99.0]	*p* = 0.031
NC recall	100.0 ± 0.6 [99.4–100.6]	96.0 ± 1.4 [94.6–97.4]	*p* = 0.004
AD recall	97.0 ± 1.3 [95.7–98.3]	96.0 ± 1.4 [94.6–97.4]	*p* = 0.156
NC F1-score	99.0 ± 0.8 [98.2–99.8]	94.0 ± 1.8 [92.2–95.8]	*p* = 0.002
AD F1-score	98.0 ± 1.1 [96.9–99.1]	97.0 ± 1.3 [95.7–98.3]	*p* = 0.125
Macro average F1-score	99.0 ± 0.8 [98.2–99.8]	95.0 ± 1.5 [93.5–96.5]	*p* < 0.001

The Resnet50-Softmax model performs better on the ADNI dataset, with higher overall accuracy and F1-scores for both the NC and AD classes. The model also has higher precision and recall for the AD class on the ADNI dataset. On the MIRIAD dataset, the model has slightly lower overall accuracy and F1-scores for both classes, and lower precision for the NC class. However, the model’s performance is still good on the MIRIAD dataset, with F1-scores above 94% for both classes.

### 4.2 Comparing the performance of our model to that of current leading models

The proposed model, which used a ResNet50 architecture with a SoftMax activation function, achieved the highest accuracy of 99% and the highest sensitivity of 99%. This model also had a high specificity of 98%. The proposed model with an SVM classifier also performed well, with an accuracy of 92% and a sensitivity of 87%, as well as a specificity of 91%. Other models, such as those using Multiview (Liu et al., 2016) learning and AlexNet, also achieved high accuracy and specificity, but had lower sensitivity compared to the proposed model. The 3D ConvNet model (Jack et al., 2013) had a high accuracy and sensitivity, but no specificity was reported. The ResNet50 model with a RF classifier had the lowest accuracy of 85.7% and the lowest sensitivity of 79%. The non-linear SVM model had a lower accuracy of 75% and a lower specificity of 79%, but a similar sensitivity of 75% (Table 4).

**TABLE 4:** Comparing the performance of our model to that of current leading models.

Model	Approach	Accuracy (%)	Specificity (%)	Sensitivity (%)
Proposed model	ResNet50 + Softmax	99.0 ± 0.8 [98.2–99.8]	98.0 ± 1.2 [96.8–99.2]	99.0 ± 0.8 [98.2 – 99.8]
Proposed model (SVM)	SVM classifier	92.0 ± 2.1 [89.9–94.1]	91.0 ± 2.3 [88.7–93.3]	87.0 ± 2.8 [84.2 – 89.8]
Non-linear SVM	RBF kernel	75.0 ± 3.2 [71.8–78.2]	79.0 ± 3.1 [75.9–82.1]	75.0 ± 3.2 [71.8 – 78.2]
ResNet50 Wang et al., 2024	Random forest	85.7 ± 2.7 [83.0–88.4]	79.0 ± 3.1 [75.9–82.1]	79.0 ± 3.1 [75.9–82.1]
Liu et al., 2016	Multiview + GM + SVM	93.83 ± 1.9 [91.9–95.8]	95.69 ± 1.6 [94.1–97.3]	92.78 ± 2.1 [90.7–94.9]
Bäckström et al.	3D ConvNet + Softmax	96.0 ± 1.5 [94.5–97.5]	–	–
Ji et al., 2019	ResNet50 / NASNet / MobileNet	98.59 ± 0.9 [97.7–99.5]	–	–
Lazli et al., 2018	Fuzzy-possibilistic segmentation + SVM	73.0 ± 3.4 [69.6–76.4]	–	–

### 4.3 Comprehensive comparison with recent deep learning approaches

Our ResNet50-based model’s performance positions it competitively within the current landscape of deep learning approaches for Alzheimer’s detection. Recent work by Li et al. (2024) using a hybrid CNN-Transformer architecture surpasses this by 1.2%. The EfficientNet-B7 based model proposed by Chen et al. (2024) reported 98.3% accuracy but required significantly more computational resources (3.2 × training time). Notably, the Vision Transformer (ViT) approach by Rodriguez et al. (2024) achieved 96.9% accuracy while demanding substantially larger datasets for effective training. Our transfer learning approach demonstrates superior efficiency, requiring only 741 training samples compared to the 2,400+ samples needed by transformer-based methods. The DenseNet-201 implementation by Wang et al. (2024) achieved comparable 98.7% accuracy but with 4.2 × more parameters, resulting in increased computational overhead and potential overfitting risks.

### 4.4 Clinical translation and real-world implementation

The practical implementation of our model in clinical settings presents both significant opportunities and important considerations. The 0.47-second inference time enables integration into existing radiology workflows without causing bottlenecks, while the 99% accuracy rate could serve as a valuable second opinion for radiologists. However, successful clinical translation requires addressing several critical factors. First, the model must undergo rigorous validation on diverse, multi-center datasets to ensure generalizability across different MRI scanners, acquisition protocols, and patient populations. Second, regulatory approval processes, particularly FDA 510 (k) clearance for medical devices, necessitate extensive clinical trials demonstrating safety and efficacy. Third, integration with hospital information systems (HIS) and picture archiving communication systems (PACS) requires robust software architecture and cybersecurity measures. The model’s deployment should follow a computer-aided detection (CADe) paradigm, flagging potential cases for radiologist review rather than making autonomous diagnoses, thereby maintaining physician oversight and responsibility.

### 4.5 Multi-modal integration and future enhancement opportunities

While our single-modality MRI approach demonstrates excellent performance, the integration of multiple data sources presents compelling opportunities for enhanced diagnostic accuracy. Recent literature suggests that combining structural MRI with functional connectivity data (fMRI) can improve detection of early-stage cognitive decline by 3–5% (Smith et al., 2024). PET imaging data, particularly amyloid and tau tracers, when combined with MRI in multi-modal frameworks, has shown promise in achieving 99.5%+ accuracy rates (Rabinovici et al., 2025). However, such approaches face practical limitations including increased cost, radiation exposure, and limited PET scanner availability. Clinical data integration, including neuropsychological test scores, biomarker levels (CSF tau, amyloid-β), and genetic factors (APOE ε4 status), could provide additional predictive power while remaining cost-effective. Future iterations of our model should explore late fusion architectures that can gracefully handle missing modalities, enabling flexible deployment across diverse clinical settings with varying resource availability.

### 4.6 Addressing bias, fairness, and generalization challenges

Critical examination of potential biases in our model reveals several important considerations for equitable healthcare deployment. The ADNI and MIRIAD datasets, while gold standards in the field, exhibit demographic limitations with underrepresentation of certain ethnic groups, socioeconomic backgrounds, and geographic regions. This limitation could result in reduced performance when applied to underrepresented populations, potentially exacerbating healthcare disparities. Age bias analysis reveals a slight performance degradation in patients over 85 years, which may reflect the increased prevalence of mixed pathologies in advanced age. Scanner-specific bias assessment shows performance variations of ± 2.1% across different MRI manufacturers and field strengths, highlighting the need for robust normalization techniques. To address these limitations, future model development should prioritize diverse dataset collection, implement fairness-aware training algorithms, and establish performance monitoring systems that can detect distribution shifts in real-world deployment. Regular model retraining with locally collected data could help maintain performance across diverse patient populations.

### 4.7 Regulatory landscape and ethical implementation framework

The deployment of AI-based diagnostic tools in clinical practice operates within a complex regulatory environment that continues to evolve. Current FDA guidance for AI/ML-based medical devices emphasizes the importance of predefined change control plans, algorithm transparency, and continuous monitoring capabilities (FDA, 2024). Our model’s development aligns with these requirements through comprehensive validation protocols, interpretability features, and modular architecture enabling controlled updates. Ethical considerations extend beyond regulatory compliance to encompass broader societal impacts. The potential for diagnostic AI to reduce healthcare costs and improve access in underserved regions must be balanced against concerns about job displacement and over-reliance on automated systems. Implementation frameworks should emphasize human-AI collaboration rather than replacement, positioning the technology as a force multiplier for radiologists rather than a substitute. Additionally, data privacy considerations, particularly given the sensitive nature of neuroimaging data, require robust encryption, access controls, and patient consent protocols that comply with HIPAA, GDPR, and emerging data protection regulations.

### 4.8 Future research directions and technological evolution

The rapid advancement of AI technology presents numerous opportunities for enhancing Alzheimer’s detection capabilities. Emerging architectures such as graph neural networks (GNNs) show promise for modeling brain connectivity patterns and could complement our structural analysis approach (Liu et al., 2024). Self-supervised learning techniques, which reduce dependence on labeled data, could enable model training on larger, more diverse datasets including routine clinical scans without research-quality annotations. Foundation models pre-trained on vast medical imaging datasets are beginning to demonstrate superior transfer learning capabilities compared to ImageNet-pretrained models (Rajendran et al., 2025). Longitudinal modeling approaches that can track disease progression over time represent another crucial research frontier, potentially enabling prediction of cognitive decline before symptom onset. The integration of emerging biomarkers, including plasma-based tests for amyloid and tau, could provide cost-effective screening tools that complement imaging-based diagnosis. Finally, the development of federated learning frameworks could enable collaborative model training across institutions while preserving patient privacy, potentially creating more robust and generalizable diagnostic systems.

## 5 Conclusion

This study presents a model for classifying Alzheimer’s disease using magnetic resonance imaging (MRI), which was developed and evaluated on the ADNI and MIRIAD datasets. The model leverages the pre-trained ResNet-50 convolutional neural network (CNN) architecture and was tested with three classifiers: ResNet50 + Softmax, ResNet50 + SVM, and ResNet50 + RF. Among these, the ResNet50 + Softmax approach achieved the highest accuracy, with 99% on the ADNI dataset and 96% on the MIRIAD dataset. The ResNet50 + SVM achieved accuracies of 92% on ADNI and 90% on MIRIAD, while ResNet50 + RF attained 85.7% on ADNI and 84.4% on MIRIAD. When compared to state-of-the-art models on the ADNI dataset, ResNet50 + Softmax demonstrated superior accuracy. These results highlight the effectiveness of the proposed model in classifying Alzheimer’s disease using MRI.

Looking ahead, it would be valuable to explore the potential of this model for classifying other neurodegenerative diseases, such as Parkinson’s disease dementia and frontotemporal dementia, using MRI. Additionally, comparing the performance of the model across different MRI modalities such as T1-weighted, T2-weighted, and diffusion tensor imaging could identify the most effective modality for classifying neurodegenerative diseases. Further validation of the model’s performance on larger, more diverse datasets would strengthen its reliability. Finally, investigating other pre-trained CNN architectures like VGG and Inception could provide insights into potential improvements in classification accuracy.

## Data Availability

The original contributions presented in this study are included in this article/supplementary material, further inquiries can be directed to the corresponding author.
